# A proteomic profiling dataset of recombinant Chinese hamster ovary cells showing enhanced cellular growth following miR-378 depletion

**DOI:** 10.1016/j.dib.2018.11.115

**Published:** 2018-11-27

**Authors:** Orla Coleman, Alan Costello, Michael Henry, Nga T. Lao, Niall Barron, Martin Clynes, Paula Meleady

**Affiliations:** a National Institute for Cellular Biotechnology, Dublin City University, Dublin 9, Ireland; b National Institute for Bioprocess Research and Training, Blackrock Co., Dublin, Ireland; c University College Dublin, Belfield, Dublin 4, Ireland

## Abstract

The proteomic data presented in this article provide supporting information to the related research article "Depletion of endogenous miRNA-378-3p increases peak cell density of CHO DP12 cells and is correlated with elevated levels of Ubiquitin Carboxyl-Terminal Hydrolase 14" (Costello et al., in press) [1]. Control and microRNA-378 depleted CHO DP12 cells were profiled using label-free quantitative proteomic profiling. CHO DP12 cells were collected on day 4 and 8 of batch culture, subcellular proteomic enrichment was performed, and subsequent fractions were analyzed by liquid chromatography tandem mass spectrometry (LC-MS/MS). Here we provide the complete proteomic dataset of proteins significantly differentially expressed by greater than 1.25-fold change in abundance between control and miR-378 depleted CHO DP12 cells, and the lists of all identified proteins for each condition.

**Specifications table**

**Table:** 

Subject area	Biotechnology
More specific subject area	Proteomics
Type of data	Excel Tables and Figure
How data was acquired	LTQ-Orbitrap XL mass spectrometer (Thermo Scientific), Progenesis QI for Proteomics (Non-linear Dynamics, Waters) and Proteome Discoverer software (Thermo Scientific)
Data format	Relative quantitation calculated and qualitative analysis
Experimental factors	Subcellular enrichment for of CHO DP12 control cells and cells depleted of microRNA-378
Experimental features	Quantitative proteomic profiling of CHO DP12 cells following microRNA-378 depletion
Data source location	Dublin, Ireland
Data accessibility	Data available in this article
Related research article	Costello, A., Coleman, O., Lao, N.T., Henry, M., Meleady, M., Barron, N. & Clynes, M. Depletion of Endogenous miRNA-378-3p Increases Peak Cell Density of CHO DP12 Cells and is Correlated with Elevated Levels of Ubiquitin Carboxyl-Terminal Hydrolase 14. Journal of Biotechnology, [1]

**Value of the data**

• This data reveals protein expression patterns associated with microRNA-378.
• Differentially expressed proteins between control and miR-378 depleted CHO cells may serve as indicators of CHO cell growth.
• This dataset reports enriched proteins from the cytosolic and membrane subcellular fractions of CHO DP12 cells.
• This data provides proteomic profiles for two time-points of CHO DP12 batch culture; exponential and stationary phase.

## 1 Data

The data presents a quantitative proteomic profiling of subcellular-enriched protein fractions from day 4 and day 8 cultures of CHO DP12 cells following microRNA-378 stable depletion. Both the cytosolic and membrane protein enriched fractions were analysed to identify significantly differentially expressed proteins between control and miR-378 depleted CHO cells (miR-378-spg) for each timepoint. Differentially expressed proteins between control and miR-378-spg cells are required to have (i) a *p-*value ≤ 0.05 on the peptide and the protein level and (ii) a minimum of 1.25-fold change in normalized abundance levels.

**Fig. 1:**
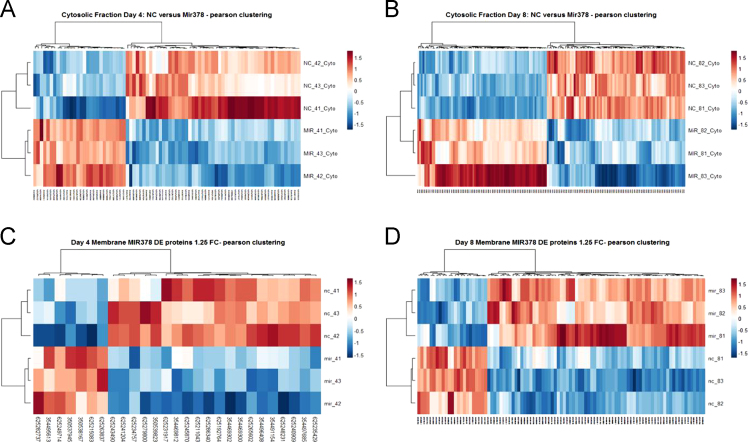
Heat maps of differentially expressed proteins in miR-378-spg CHO cells. A and B show the clustering of significantly increased and decreased proteins identified in the cytosolic enriched fraction of miR-378-spg cells for day 4 and day 8, respectively. C and D show the clustering of differentially expressed proteins identified in the membrane enriched fraction of miR-378-spg when compared to control on day 4 and day 8 of culture, respectively. The normalised abundance values of differentially expressed proteins were log2 transformed and hierarchical Pearson clustering was performed on Z-score normalised intensity values.

Table 1, Table 2, Table 3, Table 4 list the differentially expressed proteins with an increased abundance in miR-378 depleted cells when compared to control cells. Proteins with an increased abundance in miR-378-spg cells represent potential direct targets of miR-378 in CHO cells and are of most interest. Table 1, Table 2, Table 3, Table 4 report the accession number, peptide count, number of unique peptides, ANOVA *p*-value, q-value, maximum fold-change and protein name. Supplementary Table S1 presents the complete list of all differentially overexpressed and under expressed proteins for each subcellular fraction and time-point. Supplementary Table S2 presents the qualitative list of all identified proteins for each condition (control and miR-378-spg), subcellular enriched fraction (cytosolic and membrane protein enriched) and time-point (day 4 and day 8 of culture). Heat maps are shown in Fig. 1 that outlines the clustering of significantly increased versus decreased proteins in miR-378-spg cells, as compared to control cells.

**Table 1:** Mass spectrometric identification of 28 proteins from the cytosolic enriched protein fraction with ≥ 1.25-fold increase in the miR-378 depleted CHO cells on day 4 of cell culture.

**Accession**	**Peptides**	**Unique peptides**	**Anova (p)**	***Q* value**	**Max fold change**	**Protein name**
625285532	1	1	1.08E−02	2.96E−02	1.51	Splicing factor 3B subunit 3
625233305	2	2	5.27E−03	1.98E−02	1.45	Ubiquitin carboxyl-terminal hydrolase 14 isoform X4
354504493	9	9	1.24E−03	1.13E−02	1.44	6-phosphogluconate dehydrogenase, decarboxylating isoform X1
625250820	4	4	7.81E−03	2.69E−02	1.43	Copine-1 isoform X3
354500682	1	1	2.00E−02	3.88E−02	1.41	Cytochrome b5
625231502	1	1	3.25E−02	4.62E−02	1.40	Leucine-rich repeat-containing protein 47 isoform X2, partial
625204380	3	3	2.23E−04	7.06E−03	1.37	Chloride intracellular channel protein 4 isoform X1
625279800	1	1	5.08E−03	1.95E−02	1.37	Caveolin-1 isoform X1
354481364	1	1	3.55E−02	4.65E−02	1.36	crk-like protein isoform X1
625258134	1	1	9.24E−03	2.76E−02	1.35	Sulfiredoxin-1 isoform X2
625290509	1	1	3.70E−02	4.65E−02	1.34	T-complex protein 1 subunit beta isoform X2
350537945	9	9	2.38E−03	1.35E−02	1.33	Peroxiredoxin-1
625225560	2	2	5.72E−04	7.39E−03	1.32	Heterogeneous nuclear ribonucleoprotein A1 isoform X1
625260720	1	1	1.75E−02	3.77E−02	1.31	TAR DNA-binding protein 43 isoform X1
354477234	2	2	2.29E−02	3.97E−02	1.30	F-actin-capping protein subunit alpha-2 isoform X1
354502560	2	2	1.95E−03	1.35E−02	1.30	Protein DJ-1
354480001	1	1	1.29E−02	3.06E−02	1.30	T-complex protein 1 subunit delta
354495613	1	1	9.68E−03	2.82E−02	1.30	Thrombomodulin
625250988	1	1	2.32E−02	3.97E−02	1.29	Inositol-3-phosphate synthase 1 isoform X2
625224185	1	1	1.14E−02	2.99E−02	1.29	Spermidine synthase
625280088	10	10	4.60E−03	1.85E−02	1.28	Alpha-enolase isoform X3
625234360	1	1	4.51E−02	4.89E−02	1.28	Glutaredoxin-3 isoform X2
625280141	2	2	2.09E−03	1.35E−02	1.28	Cytosolic acyl coenzyme A thioester hydrolase isoform X2
625258715	1	1	4.84E−03	1.90E−02	1.26	Branched-chain-amino-acid aminotransferase, cytosolic isoform X3
625267589	1	1	3.94E−03	1.70E−02	1.26	Alpha-actinin-4 isoform X2
625233493	1	1	4.49E−02	4.89E−02	1.26	26S proteasome non-ATPase regulatory subunit 13 isoform X3
625237309	2	2	1.62E−03	1.30E−02	1.25	Adenosylhomocysteinase
625240103	2	2	1.88E−02	3.81E−02	1.25	T-complex protein 1 subunit epsilon

**Table 2:** Mass spectrometric identification of 73 proteins from the cytosolic enriched protein fraction with ≥ 1.25-fold increase in the miR-378 depleted CHO cells on day 8 of cell culture.

**Accession**	**Peptides**	**Unique peptides**	**Anova (p)**	***Q* value**	**Max fold change**	**Protein name**
625185523	1	1	3.14E−02	5.71E−03	18.24	60S ribosomal protein L26 isoform X1
625188420	1	1	3.92E−02	5.85E−03	10.57	Heterogeneous nuclear ribonucleoprotein A3 isoform X1
350539695	1	1	1.13E−02	5.34E−03	7.67	Protein disulfide-isomerase precursor
346421364	1	1	4.09E−02	5.85E−03	7.35	Calreticulin precursor
625242946	1	1	4.88E−02	6.22E−03	4.95	protein S100-A4
625223066	1	1	3.69E−02	5.85E−03	4.78	60S ribosomal protein L22 isoform X1
625195560	1	1	4.30E−02	6.00E−03	4.45	Protein disulfide-isomerase A6
625229196	2	2	4.03E−02	5.85E−03	4.13	40S ribosomal protein S6
354478978	1	1	2.80E−02	5.71E−03	3.70	protein S100-A6
625203562	1	1	2.20E−02	5.51E−03	2.84	14-3-3 protein beta/alpha
354495666	1	1	3.30E−02	5.71E−03	2.74	60S ribosomal protein L27a isoform X1
625237172	1	1	3.93E−02	5.85E−03	2.67	Cathepsin D
625272649	1	1	4.01E−02	5.85E−03	2.66	Nucleophosmin isoform X4
625191956	1	1	3.70E−02	5.85E−03	2.64	Glucosidase 2 subunit beta isoform X1
625221706	5	5	2.41E−02	5.51E−03	2.60	Alpha-enolase isoform X2
354499455	2	2	3.84E−02	5.85E−03	2.34	60S ribosomal protein L29
625193837	1	1	4.78E−02	6.22E−03	2.31	60S ribosomal protein L4 isoform X1
346986359	2	2	3.15E−02	5.71E−03	2.28	Calreticulin precursor
350539629	1	1	4.93E−02	6.22E−03	2.21	40S ribosomal protein S4
625278207	1	1	2.91E−02	5.71E−03	2.09	Transcription elongation factor B polypeptide 2 isoform X2
625234436	3	3	3.74E−02	5.85E−03	2.04	Acyl-CoA-binding protein
625290232	1	1	2.66E−02	5.51E−03	1.89	60S ribosomal protein L18 isoform X2
625194917	1	1	1.47E−02	5.34E−03	1.88	14-3-3 protein gamma
350537945	7	7	2.26E−02	5.51E−03	1.85	Peroxiredoxin-1
625225560	3	3	4.53E−02	6.15E−03	1.85	Heterogeneous nuclear ribonucleoprotein A1 isoform X1
625265794	1	1	1.13E−02	5.34E−03	1.84	60S ribosomal protein L35a
350537423	4	4	3.03E−02	5.71E−03	1.79	78 kDa glucose-regulated protein precursor
354484084	2	2	3.84E−02	5.85E−03	1.77	40S ribosomal protein S3a
354487474	5	5	3.26E−02	5.71E−03	1.75	Endoplasmin
625242866	3	3	3.26E−02	5.71E−03	1.74	Tropomyosin alpha-3 chain isoform X7
625271377	1	1	3.10E−02	5.71E−03	1.73	Peptidyl-prolyl cis-trans isomerase FKBP4 isoform X2
350538733	1	1	3.20E−02	5.71E−03	1.71	60S ribosomal protein L13
625218325	1	1	1.98E−02	5.45E−03	1.71	Y-box-binding protein 3, partial
625219233	2	2	1.46E−02	5.34E−03	1.71	Heterogeneous nuclear ribonucleoprotein D0, partial
354497356	1	1	3.08E−02	5.71E−03	1.69	ADP-ribosylation factor 3
625286340	1	1	4.66E−02	6.16E−03	1.66	Annexin A5
354507332	1	1	1.72E−02	5.34E−03	1.63	60S ribosomal protein L8
346227155	3	3	2.10E−02	5.46E−03	1.62	Elongation factor 2
625223526	1	1	1.81E−02	5.39E−03	1.59	40S ribosomal protein S3 isoform X1
625233305	3	3	5.51E−04	2.52E−03	1.58	Ubiquitin carboxyl-terminal hydrolase 14 isoform X4
625263837	1	1	1.23E−02	5.34E−03	1.55	Reticulocalbin-3 isoform X2
625204380	1	1	2.26E−02	5.51E−03	1.55	Chloride intracellular channel protein 4 isoform X1
354506476	2	2	1.53E−02	5.34E−03	1.54	Glutathione S-transferase Mu 7
625190571	3	3	2.60E−02	5.51E−03	1.47	Tropomyosin alpha-4 chain
354497863	1	1	2.57E−02	5.51E−03	1.45	RNA-binding protein FUS isoform X1
625203986	2	2	2.64E−02	5.51E−03	1.44	Peptidyl-prolyl cis-trans isomerase A
354475571	1	1	2.89E−02	5.71E−03	1.42	NSFL1 cofactor p47 isoform X1
625198438	1	1	2.48E−02	5.51E−03	1.42	Ran-specific GTPase-activating protein
625282303	1	1	1.94E−02	5.45E−03	1.40	Serine/threonine-protein kinase SMG1 isoform X3
625223520	2	2	1.48E−02	5.34E−03	1.39	Serpin H1 isoform X1
625190791	1	1	2.90E−03	4.00E−03	1.39	60S ribosomal protein L7a-like
354471594	1	1	2.06E−02	5.45E−03	1.38	Cathepsin B
625227859	3	3	1.48E−02	5.34E−03	1.37	Glutathione S-transferase Mu 6
625258134	1	1	3.04E−02	5.71E−03	1.37	Sulfiredoxin-1 isoform X2
625225201	1	1	1.69E−02	5.34E−03	1.35	Annexin A2 isoform X1
625262042	2	2	1.34E−02	5.34E−03	1.35	Heat shock protein beta-1 isoform X2
625180993	2	2	1.11E−02	5.34E−03	1.34	Eukaryotic initiation factor 4A-I-like
350540646	1	1	4.13E−02	5.85E−03	1.32	Phosphoglycerate kinase 1
625222844	3	3	2.44E−04	1.80E−03	1.32	Septin-11 isoform X1
625240830	1	1	2.66E−02	5.51E−03	1.31	Nucleoside diphosphate kinase B
625222011	1	1	4.63E−02	6.16E−03	1.30	inosine-5׳-monophosphate dehydrogenase 2 isoform X1
625289462	1	1	3.93E−02	5.85E−03	1.30	Calcium-regulated heat stable protein 1
625199022	1	1	1.31E−02	5.34E−03	1.30	m7GpppX diphosphatase
354489619	1	1	2.61E−02	5.51E−03	1.29	Isocitrate dehydrogenase [NADP] cytoplasmic
625249460	2	2	1.10E−02	5.34E−03	1.29	src substrate cortactin
625202098	1	1	4.66E−02	6.16E−03	1.29	Myosin light polypeptide 6-like
625256794	2	2	1.36E−04	1.60E−03	1.29	Fatty acid-binding protein, adipocyte
350538479	2	2	3.11E−02	5.71E−03	1.28	Tubulin beta-6 chain
625267589	5	5	3.71E−03	4.07E−03	1.28	Alpha-actinin-4 isoform X2
625206697	1	1	2.49E−02	5.51E−03	1.26	ATP-binding cassette sub-family F member 1 isoform X1
354483012	2	2	1.40E−02	5.34E−03	1.25	Heterogeneous nuclear ribonucleoprotein R
625249889	1	1	4.29E−03	4.53E−03	1.25	Caldesmon isoform X3
354465044	1	1	2.33E−02	5.51E−03	1.25	rab GDP dissociation inhibitor beta

**Table 3:** Mass spectrometric identification of 7 proteins from the membrane protein enriched fraction with ≥ 1.25-fold increase in the miR-378 depleted CHO cells on day 4 of cell culture.

**Accession**	**Peptides**	**Unique peptides**	**Anova (p)**	***Q* value**	**Max fold change**	**Protein name**
350538167	1	1	2.35E−02	3.75E−02	1.52	Calnexin precursor
354495613	2	2	2.29E−02	3.71E−02	1.37	Thrombomodulin
625263837	3	3	2.63E−03	2.00E−02	1.34	Reticulocalbin-3 isoform X2
350537945	3	3	3.71E−03	2.34E−02	1.33	Peroxiredoxin-1
625249714	1	1	3.11E−02	3.99E−02	1.31	Perilipin-4 isoform X14
625282737	1	1	4.67E−02	4.88E−02	1.31	Protein dpy-30 homolog
625215083	3	3	1.83E−02	3.38E−02	1.30	Guanine nucleotide-binding protein G(I)/G(S)/G(T) subunit beta-1 isoform X1

**Table 4:** Mass spectrometric identification of 72 proteins from the membrane protein enriched fraction with ≥ 1.25-fold increase in the miR-378 depleted CHO cells on day 8 of cell culture.

**Accession**	**Peptides**	**Unique peptides**	**Anova (p)**	***Q* value**	**Max fold change**	**Protein name**
625244585	2	2	1.10E−02	2.45E−02	2.52	Histone H2A.V isoform X2
354496412	1	1	6.56E−03	2.14E−02	2.45	Histone H1.0
354480100	5	5	2.14E−02	3.33E−02	2.17	Histone H2B type 1
354494381	1	1	1.63E−04	1.65E−02	2.12	Fibronectin isoform X1
354494231	1	1	9.56E−03	2.38E−02	2.08	High mobility group nucleosome-binding domain-containing protein 5 isoform X1
345842361	1	1	3.64E−02	4.43E−02	2.08	High mobility group protein HMG-I/HMG-Y
625206001	7	7	1.31E−02	2.76E−02	2.06	Histone H3.1-like
625285909	3	3	1.01E−02	2.38E−02	1.84	Histone H2A type 1-H-like isoform X1
625229196	1	1	4.46E−02	4.84E−02	1.81	40S ribosomal protein S6
625205207	1	1	2.86E−03	1.65E−02	1.77	rRNA 2׳-O-methyltransferase fibrillarin, partial
354480104	6	6	1.53E−02	2.89E−02	1.74	Histone H1.4 isoform X1
625289934	1	1	3.81E−02	4.46E−02	1.73	Calumenin isoform X2
350537403	1	1	2.63E−02	3.77E−02	1.68	DNA topoisomerase 2-alpha
625262546	1	1	3.48E−02	4.36E−02	1.68	Replication protein A 14 kDa subunit
625209863	1	1	1.44E−02	2.87E−02	1.67	Alpha-parvin
625234125	4	4	1.10E−02	2.45E−02	1.63	Elongation factor 1-gamma
350538167	3	3	1.78E−03	1.65E−02	1.60	Calnexin precursor
625284147	1	1	8.52E−03	2.30E−02	1.50	Legumain
350539823	1	1	4.09E−04	1.65E−02	1.50	Heat shock cognate 71 kDa protein
625204124	1	1	4.42E−02	4.82E−02	1.47	Platelet glycoprotein 4
625256908	1	1	3.23E−03	1.65E−02	1.47	Septin-2
625211254	2	2	2.82E−02	3.86E−02	1.47	Plectin isoform X1
625260069	1	1	7.33E−03	2.14E−02	1.44	14-3-3 protein epsilon isoform X2
354504493	2	2	1.54E−03	1.65E−02	1.44	6-phosphogluconate dehydrogenase, decarboxylating isoform X1
625231575	2	2	7.14E−03	2.14E−02	1.43	Eukaryotic initiation factor 4A-II isoform X1
625274484	1	1	4.37E−02	4.80E−02	1.42	Serum albumin isoform X3
625262669	1	1	3.56E−02	4.39E−02	1.42	Cellular nucleic acid-binding protein isoform X2
625243141	1	1	2.10E−02	3.32E−02	1.41	ATP-dependent RNA helicase DDX39A
625216841	1	1	6.75E−03	2.14E−02	1.41	Coronin-1B
625292335	1	1	1.81E−02	3.04E−02	1.40	High mobility group protein B2 isoform X2
354489619	1	1	7.40E−03	2.14E−02	1.40	Isocitrate dehydrogenase [NADP] cytoplasmic
625215758	1	1	2.88E−03	1.65E−02	1.39	Enoyl-CoA delta isomerase 1, mitochondrial isoform X1
354483223	1	1	2.25E−02	3.44E−02	1.39	Prolyl 4-hydroxylase subunit alpha-1 isoform X1
354467247	1	1	2.41E−03	1.65E−02	1.39	NADH dehydrogenase [ubiquinone] 1 alpha subcomplex subunit 9, mitochondrial
625284339	3	3	1.46E−02	2.87E−02	1.38	Succinate dehydrogenase ubiquinone] iron-sulfur subunit, mitochondrial isoform X2, partial
625231917	1	1	2.18E−02	3.36E−02	1.38	Guanine nucleotide-binding protein subunit beta-4
625238921	1	1	4.00E−02	4.53E−02	1.38	EH domain-containing protein 4 isoform X2
354500398	1	1	1.25E−03	1.65E−02	1.37	Ubiquitin-like modifier-activating enzyme 1 isoform X1
625190571	1	1	2.57E−02	3.74E−02	1.35	Tropomyosin alpha-4 chain
354485048	1	1	3.51E−02	4.37E−02	1.35	Polymerase I and transcript release factor
354485701	1	1	4.91E−04	1.65E−02	1.35	Stomatin-like protein 2, mitochondrial
354492573	1	1	4.19E−02	4.72E−02	1.35	Actin-related protein 3B isoform X1
354465900	2	2	1.57E−02	2.93E−02	1.35	ATP-dependent RNA helicase DDX3X isoform X1
354484391	1	1	2.57E−02	3.74E−02	1.35	14-3-3 protein zeta/delta
625190862	1	1	1.66E−02	2.97E−02	1.34	NADH dehydrogenase [ubiquinone] 1 alpha subcomplex subunit 10, mitochondrial isoform X1
625240103	1	1	4.16E−03	1.83E−02	1.34	T-complex protein 1 subunit epsilon
625188420	1	1	4.22E−02	4.73E−02	1.33	Heterogeneous nuclear ribonucleoprotein A3 isoform X1
625211596	1	1	6.89E−04	1.65E−02	1.33	60S ribosomal protein L7 isoform X1
625248231	1	1	2.96E−03	1.65E−02	1.33	ADP/ATP translocase 1 isoform X2
625251833	1	1	2.86E−02	3.86E−02	1.32	Hydroxymethylglutaryl-CoA lyase, mitochondrial isoform X3
350540646	2	2	1.59E−02	2.93E−02	1.32	Phosphoglycerate kinase 1
625254434	1	1	6.32E−03	2.14E−02	1.32	Superoxide dismutase [Mn], mitochondrial isoform X2
625249635	1	1	5.33E−03	2.06E−02	1.31	lon protease homolog, mitochondrial
625208910	1	1	4.97E−02	5.03E−02	1.30	Septin-7 isoform X1
354486011	1	1	4.29E−02	4.74E−02	1.30	Acyl-coenzyme A thioesterase 1 isoform X1
625213146	1	1	9.70E−03	2.38E−02	1.29	Integrin beta-1 isoform X1
625232358	3	3	4.03E−04	1.65E−02	1.29	Lipoprotein lipase isoform X1
625279800	1	1	4.90E−02	5.00E−02	1.28	Caveolin-1 isoform X1
625235290	1	1	4.73E−02	4.89E−02	1.28	Peroxiredoxin-5, mitochondrial
625215083	2	2	1.26E−03	1.65E−02	1.28	Guanine nucleotide-binding protein G(I)/G(S)/G(T) subunit beta-1 isoform X1
346986359	3	3	2.62E−03	1.65E−02	1.27	Elongation factor 1-alpha 1
625201330	1	1	1.81E−02	3.04E−02	1.27	Cell division control protein 42 homolog
625288359	2	2	1.73E−02	3.01E−02	1.27	dephospho-CoA kinase domain-containing protein
552953713	1	1	1.43E−02	2.87E−02	1.26	40S ribosomal protein S7
354482483	1	1	2.73E−02	3.82E−02	1.26	vimentin
625243995	2	2	3.76E−02	4.46E−02	1.26	Leucine-rich PPR motif-containing protein, mitochondrial isoform X2
625184898	1	1	3.26E−02	4.24E−02	1.25	39S ribosomal protein L12, mitochondrial isoform X1
625236680	1	1	1.11E−02	2.45E−02	1.25	60 kDa heat shock protein, mitochondrial
625183009	2	2	1.37E−02	2.85E−02	1.25	Triosephosphate isomerase isoform X1
354486540	2	2	2.91E−03	1.65E−02	1.25	Hydroxymethylglutaryl-CoA synthase, mitochondrial
625224152	1	1	2.50E−02	3.72E−02	1.25	Nuclear body protein SP140-like isoform X1
625291524	1	1	3.79E−02	4.46E−02	1.25	Mitochondrial import inner membrane translocase subunit Tim13 isoform X3, partial

## 2 Experimental design, materials and methods

### 2.1 Subcellular protein extraction and in-solution protein digestion

Triplicate biological samples for control and miR-378 depleted cells were collected on day 4 and day 8 of batch cultures. Subcellular protein enrichment was achieved using the Mem-Per Plus Membrane protein extraction kit (#89842, Thermo Fisher Scientific) which yielded a cytosolic and membrane protein enriched fraction. Protein concentration was determined using the QuickStart Bradford assay (Bio-rad). Equal concentrations (100 µg) of protein from each sample were purified and trypsin digested for mass spectrometry using the filter-aided sample preparation method as previously described [2]. The resulting peptide samples were purified using Pierce C18 spin columns then dried using vacuum centrifugation and suspended in 2% acetonitrile and 0.1% trifluoracetic acid in LC grade water prior to LC-MS/MS analysis.

### 2.2 Label-free liquid chromatography mass spectrometry

Quantitative label-free liquid-chromatography mass spectrometry (LC–MS/MS) analysis of mir-378-spg and NC-spg membrane and cytosolic fractions from day 4 and day 8 was carried out using a Dionex UltiMate™ 3000 RSLCnano system (Thermo Fisher Scientific) coupled to a hybrid linear LTQ Orbitrap XL mass spectrometer (Thermo Fisher Scientific). LC-MS/MS methods were applied as previously described [3]. A 5 μL injection of each sample was loaded onto a C18 trapping column (PepMap100, C18, 300 μm × 5 mm; Thermo Fisher Scientific). Each sample was desalted for 5 min using a flow rate of 25 μL/min with 2% ACN, 0.1% TFA before being switched online with the analytical column (PepMap C18, 75 μm ID × 250 mm, 3 μm particle and 100 Å pore size; (Thermo Fisher Scientific)). Peptides were eluted using a binary gradient of Solvent A (2% ACN and 0.1% formic acid in LC grade water) and Solvent B (80% ACN and 0.08% formic acid in LC grade water). The following gradient was applied; 6–25% solvent B for 120 min and 25–50% solvent B in a further 60 min at a column flow rate of 300 nL/min. Data was acquired with Xcalibur software, version 2.0.7 (Thermo Fisher Scientific). The LTQ Orbitrap XL was operated in data-dependent mode with full MS scans in the 400–1200 m/z range using the Orbitrap mass analyser with a resolution of 30,000 (at m/z 400). Up to three of the most intense ions (+1, +2, and +3) per scan were fragmented using collision-induced dissociation (CID) in the linear ion trap. Dynamic exclusion was enabled with a repeat count of 1, repeat duration of 20 s, and exclusion duration of 40 s. All tandem mass spectra were collected using a normalized collision energy of 32%, and an isolation window of 2 m/z with an activation time of 30 ms.

### 2.3 Quantitative label-free LC-MS/MS data analysis

Protein identification was achieved using Proteome Discoverer 2.1 with the Sequest HT and MASCOT algorithm followed by Percolator validation [4] to apply a false-discovery rate < 0.01. Data was searched against the NCBI Chinese Hamster (*Cricetulus griseus*) protein database containing 44,065 sequences (fasta file downloaded November 2015). The following search parameters were used for protein identification: (1) precursor mass tolerance set to 20 ppm, (2) fragment mass tolerance set to 0.6 Da, (3) up to two missed cleavages were allowed, (4) carbamidomethylation of cysteine set as a static modification and (5) methionine oxidation set as a dynamic modification. The complete lists of all identified proteins from the cytosolic and membrane enriched fractions of day 4 and day 8 cell cultures of the control (NC378-spg) and miR-378-spg are provided in Supplementary Table S2.

Quantitative label-free data analysis was performed using Progenesis QI for Proteomics (version 2.0; Nonlinear Dynamics, a Waters company) as described by the manufacturer (www.nonlinear.com). To counteract potential drifts in retention time a reference run was assigned to which all MS data files were aligned. The triplicate samples from the two experimental groups (NC-378-spg and miR-378-spg) were set up for differential analysis and label-free relative quantitation was carried out after peak detection, automatic retention time calibration and normalisation to account for experimental variation. The experimental analyses performed compared the three biological replicates for control cells to miR-378-spg triplicates for each timepoint and subcellular fraction collected. The following settings were applied to filter peptide features (1) peptide features with a one-way ANOVA p-value < 0.05 between experimental groups, (2) mass peaks with charge states from +1 to +3 and (3) greater than one isotope per peptide. The normalised data is transformed prior to statistical analysis, using an arcsinh transformation to meet the assumptions of the one-way ANOVA test. A mascot generic file (mgf) was generated from all exported MS/MS spectra which satisfied the peptide filters, the mgf was used for peptide and protein identification in Proteome Discoverer. Protein identifications were imported into Progenesis and considered differentially expressed if they passed the following criteria: (i) a protein one-way ANOVA p-value <0.05 and (ii) a ≥1.25-fold change in relative abundance between the two experimental groups. All differentially expressed proteins identified between NC378-spg and miR-378-spg cells are reported in Supplementary Table S1.

Heatmaps illustrating protein abundances for statistically significant and differentially expressed proteins were designed using ggplot2 in R-studio. The normalised abundance values of differentially expressed proteins were determined using Progenesis QI for Proteomics and were loaded as a txt file into R-studio and the data was log2 transformed. Hierarchical Pearson clustering was then performed on Z-score normalised intensity values by clustering both samples and proteins.
