# Supplementary material for: A proteomic profiling dataset of recombinant Chinese hamster ovary cells showing enhanced cellular growth following miR-378 depletion
Source: Data Brief. 2018 Nov 27;21:2679–88. doi: 10.1016/j.dib.2018.11.115 (PMC6290246; doi:10.1016/j.dib.2018.11.115)
Supplement: Supplementary file 1 — Transparency document [file mmc1.pdf]

Data-in-brief article

Title: *Label-free quantitative proteomic profiling of miR-378 depleted CHO cells showing enhanced cell growth*

Authors: Orla Coleman[1]*, Alan Costello[1]*, Michael Henry[1], Nga T. Lao[1], Niall Barron[2,3], Martin Clynes[1]* and Paula Meleady[1]*
* These authors contributed equally

Affiliations:

[1]National Institute for Cellular Biotechnology, Dublin City University, Dublin 9, Ireland
[2]National Institute for Bioprocess Research and Training, Blackrock, Co. Dublin , Ireland
[3]University College Dublin, Belfield, Dublin 4, Ireland

Contact email: orla.coleman2@mail.dcu.ie

Conflict of Interest:

All of the authors declare no conflict of interest
